# The Emission of the Floral Scent of Four *Osmanthus fragrans* Cultivars in Response to Different Temperatures

**DOI:** 10.3390/molecules22030430

**Published:** 2017-03-08

**Authors:** Jianxin Fu, Dan Hou, Chao Zhang, Zhiyi Bao, Hongbo Zhao, Shaoqing Hu

**Affiliations:** 1 Department of Ornamental Horticulture, School of Landscape Architecture, Zhejiang Agriculture and Forestry University, Lin’an 311300, China; fujianxin2008@sohu.com (J.F.); zhangchao24804@gmail.com (C.Z.); bao99928@188.com (Z.B.); 2 State Key Laboratory of Subtropical Silviculture, Zhejiang Agriculture and Forestry University, Lin’an 311300, China; houxiaohou15@aliyun.com; 3 College of Civil Engineering and Architecture, Zhejiang Sci-Tech University, Hangzhou 310018, China; 13600512490@163.com

**Keywords:** floral scent, *Osmanthus fragrans*, temperature, volatile organic compounds, SPME, GC-MS

## Abstract

Floral scent is an important part of volatile organic compounds (VOCs) emitted from plants, and is influenced by many environmental and endogenous factors. To investigate the influence of temperature on the emission of the floral scent of *Osmanthus fragrans*, the number of chemical compounds and their relative release amounts from four cultivars of *O. fragrans* under different temperature treatments, were identified using the solid-phase microextraction (SPME) technique and gas chromatography-mass spectrometry (GC-MS) in this study. Results revealed that the numbers and release amounts of floral scent components were significantly influenced by different temperatures, and depend on different cultivars and different types of compounds. Overall, most cultivars had the largest number of chemical compounds in 19 °C and the numbers of chemical compounds decreased with the increase or decrease in the temperature. Alcohols and ketones were the two main kinds of compounds responding to temperature change. The response of a specific chemical compound to temperature change was different in four cultivars. Generally, linalool, α-ionone, β-ionone, and γ-decalactone accounted for the highest proportion in the nine main compounds, and changes of these four chemical compounds to different temperatures had obvious contributions to the floral scent of *O. fragrans*. The results obtained provide evidence that temperatures can greatly influence the emission of floral scent.

## 1. Introduction

Plant organs, such as flowers, fruits, leaves and roots, can produce and emit a large variety of volatile organic compounds (VOCs) that are useful in their interactions with their immediate environment. At present, more than 1000 VOCs have been reported to be emitted from vegetative parts as well as flowers [1]. Most of these VOCs can be assigned to the following classes: terpenoids, phenylpropanoids, and benzenoids, fatty acid derivatives, and amino acid derivatives [2,3]. As an important part of VOCs emitted from plants, a floral scent plays a significant role in many eco-physiological processes [4]. Floral scents are not only responsible for attraction of butter flies, moths, and bats for pollination, but also act as semiochemicals for the communication with neighboring plants [5]. Moreover, some floral scent compounds are usually distillated to obtain essential oils for further applications such as aromatherapy, perfume, flavoring, and food additives [5].

Many studies have shown that the environmental factors such as temperature [6,7,8,9,10,11,12,13], light [8,14], atmospheric CO_2_ concentration [12,15,16,17], agricultural practices (e.g., irrigation and fertilization) [18], and endogenous factors including circadian rhythms [19,20,21], leaf age [16,22,23], floral developmental stages [24,25] and plant age [26], are responsible for the production and emission rates of floral scents. In the past, researchers have focused on the effect of temperature on the VOCs emitted from plant leaves and fruits. With the enhancement of temperature, the volatile concentrations of apples increase, although production rate is reduced above 32 °C [27]. The emission rate of five monoterpenes shows positive correlation with leaf temperature between 20 °C and 46 °C in *Pinus elliottii* [28]. Isoprene emission from plant leaves increases with temperature and reaches the highest release rate at 40 °C [29]. Terpenoids and indole emissions increase with temperature to an optimum between 22 and 27 °C and thereafter decrease in corn plants [30]. Analysis of volatiles from blended leaves in sweet basil by gas chromatography-mass spectrometry (GC-MS) showed that the plants grown at 25 °C contained significantly more volatile oils than those grown at 15 °C [31].

Since the floral scent plays a key role in plant pollination and plant defense response, more and more attention has been paid to the effect of temperature on floral scent emission. It has been found that the total release amount of floral scent are greatly affected by the different temperatures in *Trifolium repens* [32], *Petunia axillaris* [33], and *Lilium* ‘Siberia’ [8]. The total endogenous amount of scent components decreased as the temperature increased, the total emission showing a peak at 30 °C in *Petunia axillaris* [33]. The results in *Lilium* ‘Siberia’ revealed that the numbers and release amounts of floral scent components were significantly influenced by temperature, and 30 °C resulted in the highest numbers and release amounts of the floral scent components [8].

**Figure 1:**
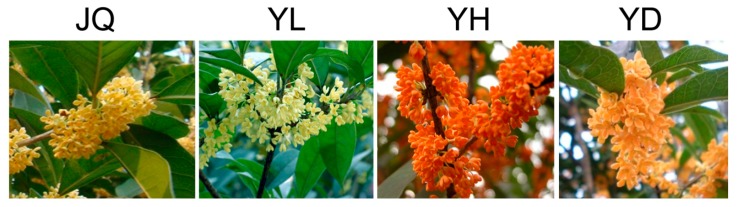
The floral morphology of four cultivars “Jin Qiugui” (JQ), “Yu Linglong” (YL), “Yanhong Gui” (YH), and “Yingye Dangui” (YD) of *Osmanthus fragrans* in this study.

*Osmanthus fragrans* belongs to the Oleaceae family and is one of the ten most famous flowers in China. They are evergreen trees and shrubs, grown as ornamental plants for their attractive foliage and fragrant edible flowers. Their flowers are usually distillated to obtain essential oils for further applications such as aromatherapy, haute couture perfumes, flavoring, food additives, and consumer products, such as soaps, shampoos, and detergents just because their particular fragrance. Floral fragrance is a desirable character for ornamental plants [34]. Because of the unique and remarkable scent, much research has focused on the identification of the components of the floral scent of *O*. *fragrans* [35,36,37]. A wide variety of volatile compounds were identified in different cultivars of *O*. *fragrans*, including α-ionone, β-ionone, linalool, *cis*- and *trans*-linalool oxide, γ-decalactone, and so on. In previous studies, these volatile compounds were greatly influenced by different temperatures in other ornamental plants *Petunia axillaries* [33], *Lilium* ‘Siberia’ [8], and different cultivars of *Petunia* × *hybrida* [7]. Since environmental temperature could greatly change in nature during the floral opening of *O*. *fragrans*, the release of floral scent is supposed to be influenced, which motivates us to gain knowledge of the relationships between floral scents and the surrounding temperatures. In this study, the floral scent emitted from four cultivars, “Yu Linglong” (YL) from the Albus group, “Jin Qiugui” (JQ) from the Luteus group, and “Yanhong Gui” (YH) and “Yingye Dangui” (YD) from the Aurantiacus group (Figure 1), of *O*. *fragrans* was collected by dynamic headspace at different levels of temperatures and further analyzed by using the solid-phase microextraction (SPME) technique and GC-MS technique. The numbers of chemical compounds and relative release amounts were subsequently identified to investigate the influence of temperature on the emission of floral scent.

## 2. Results

### 2.1. Influence of Temperature on the Number of Chemical Compounds Emitted from Osmanthus fragrans

In total, 53 chemical compounds were identified (Table 1 and Table 2) in this study. As shown in Table 3, with the treatment of medium temperature (19 °C), 29 chemical compounds were identified in YL, which was the highest of the four cultivars, while only 23 chemical compounds were detected in YH. Many of these compounds belong to the class of alkanes, but just one is an alkene compound. High-temperature treatment (32 °C) reduced the number of compounds of alkanes and ketones in JQ, YL, and YD (Table 3) and the number of compounds of alcohols in JQ and YL; therefore, the number of total chemical compounds in these cultivars, was respectively reduced by 25.92%, 17.24%, and 33.33%, compared with the treatment of 19 °C. However, four additional alkane compounds (4,6-dimethyldodecane, 3-methyltetradecane, 2-methylheptadecane, and pentadecane) were found in YH with high-temperature treatment. Generally, the number of ketones was reduced in four cultivars with high-temperature treatment.

Low temperature (15 °C) treatment increased the number of alkenes and decreased the number of alkanes in JQ, YL, and YD, but had no significant effect on those two kinds of chemical compounds in YH. Moreover, the treatment of 15 °C had no significant effect on the number of alcohols and esters in four cultivars. In YL, the number of ketones was reduced by more than half with the treatment of 15 °C, while the number of ketones was similar in the three other cultivars with the treatments of 15 °C and 19 °C. Under lower temperature treatment (12 °C), the number of total chemical compounds in YL and YD was much lower than that with treatments of 19 °C; however, for JQ and YH, the number of total chemical compounds was similar to that with treatments of 19 °C. Generally, the treatment of 12 °C had no significant effect on the number of alcohols and alkenes among the four cultivars. The treatment of 12 °C greatly decreased the number of ketones in JQ, YL, and YH, and the number of alkanes in YL and YD.

### 2.2. Influence of Temperature on the Relative Content of Chemical Compounds Emitted from Osmanthus fragrans

**Figure 2:**
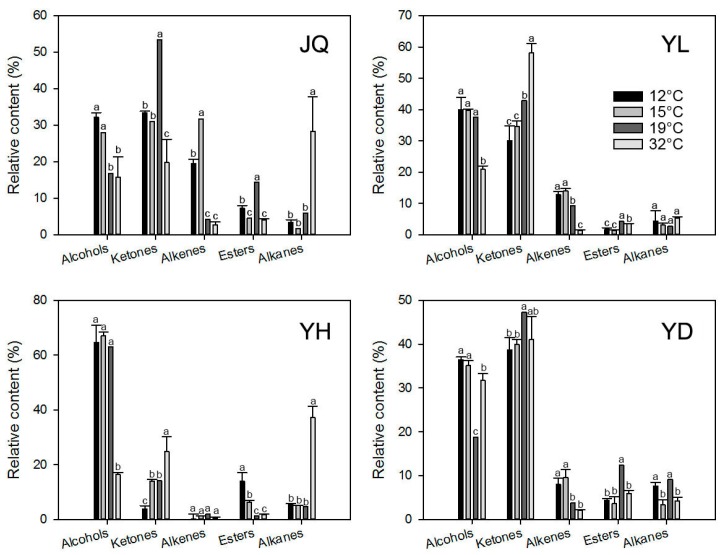
Release amounts of different volatile categories in floral scent emitted from four cultivars of *Osmanthus fragrans* at different levels of temperature (12, 15, 19, and 32 °C). Error bars indicate SE (*n* = 3). Letters indicate significant differences (*p* < 0.05 by least significant difference) among the release amounts at different temperature treatments.

The relative release content of volatile compounds in the floral scent of *O. fragrans* at different temperature levels was also compared. Different temperatures significantly influenced the relative release content of different compound categories (*p* < 0.05; Figure 2). Compared to 19 °C, the relative release content of alcohols significantly increased in JQ and YD with the treatments of 12 and 15 °C; however, that in YL and YH was not changed under the treatments of 12 and 15 °C. The relative release content of alcohols with the treatment of 32 °C significantly decreased in YL and YH. Low temperatures of 12 and 15 °C decreased the relative release content of ketones in JQ and YD, but high temperatures of 32 °C reduced the relative release content of ketones in JQ. Similarly, in YL, the relative release content of ketones decreased when the temperature decreased to 12 and 15 °C, but increased when the temperature increased to 32 °C. In YH, the relative release content of ketones did not change with the treatments of 15 °C, but decreased with treatment of 12 °C and increased with treatment of 32 °C. The relative release content of alkenes significantly increased in most cultivars except in YH when the temperature decreased to 12 or 15 °C; however, it decreased in YL or did not change at all in the three other cultivars when the temperature increased to 32 °C. The relative release content of esters decreased whenever the temperature decreased to 12 and 15 °C or increased to 32 °C in JQ, YL, and YD. However, in YH, it increased when the temperature decreased to 12 and 15 °C and did not change at all when the temperature increased to 32 °C. Alkanes in all cultivars were not changed when the temperature decreased to 12 and 15 °C, but they significantly increased in JQ and YH when the temperature increased to 32 °C.

### 2.3. Influence of Temperature on The Relative Content of Main Chemical Compounds Emitted from Osmanthus fragrans

**Figure 3:**
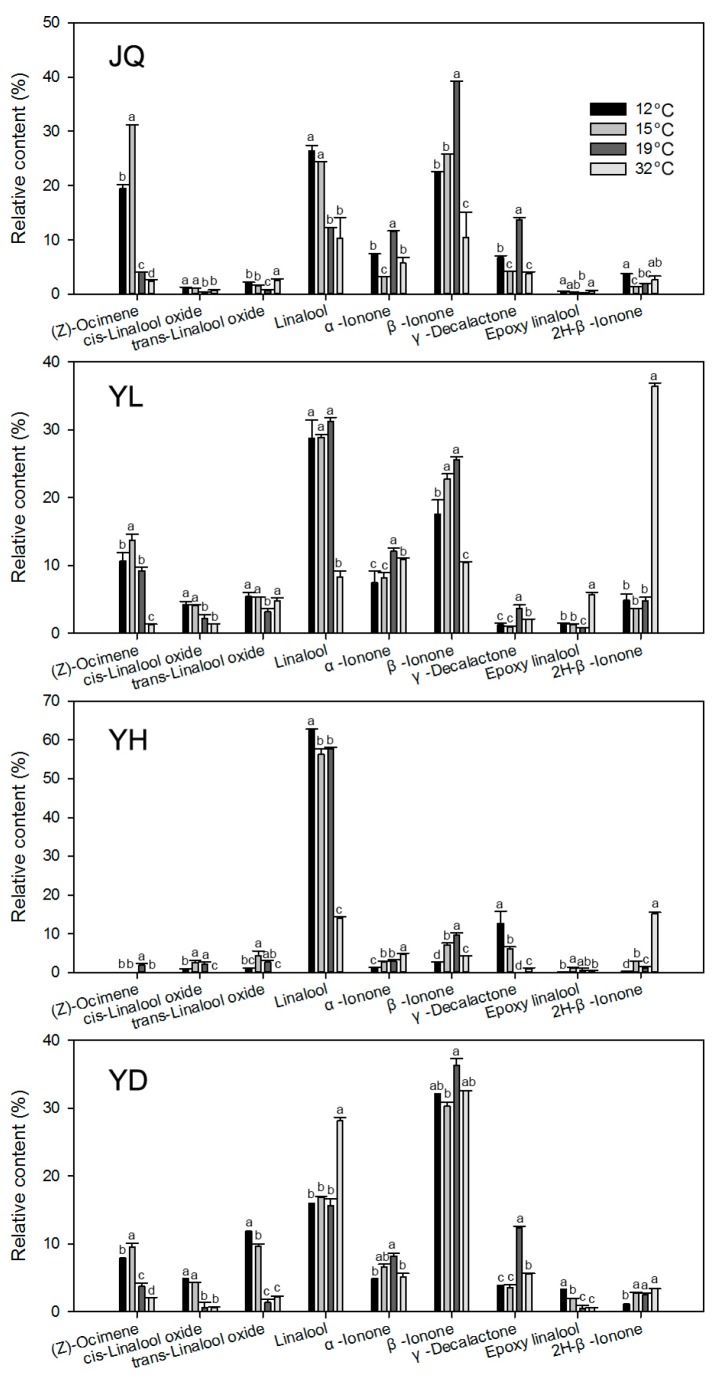
Release amounts of nine volatile components in floral scent emitted from four cultivars of *Osmanthus fragrans* at different levels of temperature (12, 15, 19, and 32 °C). Error bars indicate SE (*n* = 3). Letters indicate significant differences (*p* < 0.05 by least significant difference) among the release amounts at different temperature treatments.

We also investigated the relative release amount of nine main chemical compounds that are beyond 1% in four cultivars of *O. fragrans* (Figure 3). The same chemical compound showed different changes to the same temperature change in different cultivars. For example, the relative release amount of (*Z*)-ocimene in most cultivars increased but decreased in YH when the temperature fell to 12 and 15 °C, which decreased in all cultivars with the treatment of 32 °C. Except for YH, the relative release amount of *cis*-linalool oxide and *trans*-linalool oxide increased with the fall of temperature to 12 and 15 °C and had no significant change or changed little with the increase in temperature to 32 °C. Only in JQ did the relative release amount of linalool significantly increase when the temperature fell to 12 and 15 °C. When the temperature increased to 32 °C, the relative release amount of linalool showed three different patterns: an increase in YD, a decrease in YL and YH, and no change in JQ. Whenever the temperature increased or decreased, the relative release amount of both α-ionone and β-ionone significantly decreased in all cultivars except for YH. The relative release amount of γ-decalactone significantly decreased in JQ, YL, and YD whenever the temperature increased or decreased; however, that significantly increased in YH whenever the temperature increased or decreased. The relative release amount of epoxy linalool increased in YL and YD when the temperature decreased to 12 and 15 °C, and it increased in JQ and YL with the treatment of 32 °C. The relative release amounts of 2*H*-β-ionone significantly increased in YL and YH when the temperature increased to 32 °C.

## 3. Discussion

Temperature is an important environmental factor to influence the emission of volatile compounds from plants [8,13,33]. In this study, the emissions of floral scent from four cultivars of *O. fragrans* treated with different levels of temperatures were investigated. Generally in most cultivars, the treatment of 32 °C resulted in lower numbers of the chemical volatile compounds than 19 °C (Table 3). Similarly, compared with 30 °C, the number of chemical compounds decreases with the increase of the temperature to 40 °C in *Lilium* ‘Siberia’ [8]. It was also found in *Petunia axillaris* [33] and Mediterranean plants [38] that the amount of floral scent greatly decreases with the enhancement of temperature.

Moreover, a high temperature negatively influences the release of chemical volatile compounds [7,8,33,38]. A high temperature of 40 °C significantly decreases most volatile components of *Lilium* ‘Siberia’, especially *trans*-ocimene, α-ocimene, and linalool [8]. In *Petunia axillaris*, 35 °C reduced not only release the amount of floral scent compounds, but also their endogenous levels compared with the other lower temperatures [33]. A high temperature (32 °C) shows influences similar to *Lilium* ‘Siberia’ and *Petunia axillaris* in *O. fragrans*, which was found to greatly decrease the relative release amount of α-ionone, β-ionone, γ-decalactone, and (*Z*)-ocimene in most cultivars (Table 2 and Figure 3). Two potential pathways contributed to the change in floral scent at different temperatures. On the one hand, the biosynthesis processes regulated by their synthesis enzymes. On the other hand, vaporization was also an important factor. It has been reported that temperature influences both metabolic and vaporization processes of the floral scent emission in *Petunia axillaris* [33]. What is more, in *Petunia × hybrid*, increasing ambient temperature leads to a decrease in phenylpropanoid-based floral scent production, which could be attributed to the downregulation of scent-related structural genes’ expression and the upregulation of a negative regulator of scent. A high temperature may downregulate volatile compound-related gene expression and reduce their enzyme activities, which would result in a lower release amount of many chemical volatile compounds in *O. fragrans*.

Downregulation of high temperature did not occur in the release of all chemical volatile compounds. For instance, 32 °C significantly increased the relative release amount of 2*H*-β-ionone in YL and YH (Table 2 and Figure 3), which generated an increase in the release amount of ketones, although 32 °C reduced the emission of other ketone compounds such as α-ionone and β-ionone in these two cultivars. A hypothesis was raised that gene expression related to some chemical volatile compounds such as 2*H*-β-ionone was probably upregulated by 32 °C. In addition, Farré-Armengol et al. revealed that the response of floral emissions to temperature differed among species and among different compounds within the species, after measuring the temperature responses of floral emissions of various common species of Mediterranean plants [38], suggesting that the response of floral emissions to temperature might differ among cultivars of *O. fragrans*.

As for lower temperatures, 12 °C resulted in lower numbers of the chemical volatile compounds than 19 °C in all four cultivars (Table 3), while 15 °C decreased numbers of the chemical volatile compounds in YL and YD and slightly affected numbers in JQ and YH (Table 3), which was similar to the results in *Lilium* ‘Siberia’ [8] that numbers of chemical compounds decrease with the decrease in temperature from 30 to 10 °C. In additional, the release amounts of ketone and ester compounds was significantly lower with the lower temperature treatments (10 and 20 °C) [8]. In accordance with the results of *Lilium* ‘Siberia’ lower temperatures (12 and 15 °C) resulted in lower release amounts of volatile categories ketones and esters in most cultivars (Figure 2). To be specific, the release amounts of α-ionone and β-ionone from ketones, and γ-decalactone from esters were greatly reduced with the lower temperature treatments in most cultivars (Figure 3). However, the release amounts of some volatile compounds were greatly increased with the lower temperature treatments, such as (*Z*)-ocimene, *cis*-linalool oxide, and *trans*-linalool oxide (Figure 3). As mentioned above, the release of a certain volatile compound is complex, which depends on the combined action of the biosynthesis processes and vaporization. Based on the endogenous levels of floral scent compounds at different temperatures in *Petunia axillaris*, the endogenous levels of iso-eugenol, benzyl benzoate, benzyl alcohol, and 2-phenylethanol gradually increases with the decrease in temperature from 30 to 20 °C [33]. The release amounts of iso-eugenol and benzyl benzoate gradually decreases with the decrease in temperature from 30 to 20 °C; however, the release amounts of benzyl alcohol and 2-phenylethanol increases first and then decreases from 30 to 20 °C [33], indicating that the increase in the endogenous amount by the decrease in temperature could be recovered by an increase in vapor pressure, which probably corresponds to the emission ratio.

## 4. Materials and Methods

### 4.1. Plant Material

Five-year-old potted plants of four *O. fragrans* cultivars, “Yu Linglong” (YL) from Albus group, “Jin Qiugui” (JQ) from Luteus group, “Yanhong Gui” (YH) and “Yingye Dangui” (YD) from Aurantiacus group (Figure 1), grown under the field condition in the germplasm repository of Zhejiang Agriculture and Forestry University, were employed as materials. When the developmental stages of flowers reached to Linggeng stage (the inflorescence burst through bracts and the florets closely crowded), potted plants were transported to the laboratory for treatments.

### 4.2. Temperature Treatments

Climate chambers were employed to provide different temperatures for the treatments. Treatments with low temperatures of 12 and 15 °C, medium temperature of 19 °C, and high temperature of 32 °C were used in this study. For each cultivar, three potted plants were cultured in a climate chamber with certain temperature (12, 15, 19, or 32 °C), and 12 potted plants were used for temperature treatments in total. Light intensity was maintained at 90–108 μmol·m^−2^·s^−1^ with a relative humidity of 80% in each climate chambers. Potted plants were continuously treated until the flowers were fully opened.

### 4.3. Floral Scent Collection

The samples were collected when the flowers were fully opened. A portion of 0.4 g of the entire flowers from all treatments were respectively detached and placed into brown glass vials (20 mL) capped with plastic caps having a polytetrafluoroethylene septum. Floral volatiles were collected using the solid-phase microextraction (SPME) method [24]. A fiber of 100 μm poly dimethyl-siloxane (PDMS, Supelco, Bellefonte, PA, USA) was used following the methods used in tree peony [24]. After 15 min equilibration, the aged PDMS fiber was exposed to each sample for 40 min and was then transferred into the injection port of the GC-MS systems. Before extraction, the fiber was aged for 1 h at 230 °C. Three biological replicate samples were collected from each treatment.

### 4.4. GC-MS Analysis

The samples were analyzed by gas chromatography-mass spectrometry (GC-MS QP2010 Plus, SHIMADZU, Kyoto, Japan) fitted with a gas chromatograph column Restek Rtx-Wax (30 m × 0.25 mm). Helium (99.999%) was used as the carrier gas at a flow rate of 1.0 mL·min^−1^. The column pressure was 49.5 Pa, and the desorption time was 1 min. The initial oven temperature of the column was maintained at 40 °C for 5 min and then ramped to 250 °C at 5 °C·min^−1^, and maintained for 5 min. The temperature of the ion source and interface temperature were 230 °C and 250 °C, respectively. The mass spectrometer was operated in electron ionization (EI) mode, and the ionization potential was 70 eV, scanning the range of 33–650 amu.

The identification of the compounds was done based on the retention index (RI) by comparison of the mass spectra with the NTST08 and NTST08s databases through a G1701DA ChemStation (Agilent, Palo Alto, CA, USA). The constituents were further confirmed by comparing with the published references [39,40,41,42,43,44]. Retention indices (RI) were calculated using retention times of n-alkanes that had been injected to the same instrument and under the same chromatographic conditions. Quantitative analysis in percent was performed by peak area normalization measurements [24,45,46,47].

### 4.5. Statistical Analysis

The statistical analysis was performed by one-way analysis of variance (ANOVA), using SPSS software version 18.0 (SPSS Inc., Chicago, IL, USA). Data are expressed as average ± standard errors of three biological replicates. Least significant difference (LSD) test was employed and differences of *p* < 0.05 were considered significant. 

## Figures and Tables

**Table 1:** All identified volatile compounds in this study.

No.	Volatile Category	Compound Name	Code
**1**	Alcohols	*cis*-Linalool oxide	A1
**2**		*trans*-Linalool oxide	A2
**3**		Linalool	A3
**4**		3,7-Dimethyl-1,5,7-octatrien-3-ol	A4
**5**		Epoxy linalool	A5
**6**		4-(2,6,6-Trimethyl-cyclohex-1-enyl)-butan-2-ol	A6
**7**		Cedrol	A7
**8**		2,6-Dimethyl-1,7-octadiene-2,6-diol	A8
**9**		2-(4-Methoxyphenyl)ethanol	A9
**10**	Ketones	6-Methyl-5-hepten-2-one	B1
**11**		2,2,6-Trimethyl-6-ethenyldihydro-2*H*-pyran-3(4*H*)-one	B2
**12**		4-(2,6,6-Trimethyl-2-cyclohexen-1-ylidene)-2-butanone	B3
**13**		2*H*-α-Ionone	B4
**14**		2*H*-β-Ionone	B5
**15**		α-Ionone	B6
**16**		Geranyl acetone	B7
**17**		β-Ionone	B8
**18**		4-(2,2,6-Trimethyl-7-oxabicyclo[4.1.0]hept-1-yl)-3-buten-2-one	B9
**19**		Pseudo ionone	B10
**20**		6-Pentyl-2*H*-pyran-2-one	B11
**21**	Alkenes	(*E*)-Ocimene	C1
**22**		(*Z*)-Ocimene	C2
**23**		2,2,4,6,6-Pentamethyl-3-heptene	C3
**24**		2,2,4,10,12,12-Hexamethyl-7-(3,5,5-trimethylhexyl)-6-tridecene	C4
**25**		7-Tetradecene	C5
**26**		α-farnesene	C6
**27**	Esters	(*Z*)-3-Hexenyl butanoic acid ester	D1
**28**		(*Z*)-4-Hexen-1-yl butanoic acid ester	D2
**29**		*cis*-3-Hexenyl-α-methylbutyrate	D3
**30**		*cis*-3-Hexenyl *n*-valeric acid ester	D4
**31**		γ-Decalactone	D5
**32**	Alkanes	Tridecane	E1
**33**		3,7-Dimethyldecane	E2
**34**		Tetradecane	E3
**35**		4,6-Dimethyldodecane	E4
**36**		3-Methyltetradecane	E5
**37**		Pentadecane	E6
**38**		2-Methylheptadecane	E7
**39**		3-Methylpentadecane	E8
**40**		Hexadecane	E9
**41**		Nonadecane	E10
**42**		2,6,10,14-Tetramethylpentadecane	E11
**43**		8-Hexylpentadecane	E12
**44**		2,6,10,14-tetramethylhexadecane	E13
**45**		Heptadecane	E14
**46**		Octadecane	E15
**47**		Pentacosane	E16
**48**		Heneicosane	E17
**49**		Eicosane	E18
**50**		Hexatriacontane	E19
**51**		Dotriacontane	E20
**52**		Tetrapentacontane	E21
**53**		Tetracontane	E22

**Table 2:** Average relative content of each volatile compounds response to different temperatures. Code of each volatile compound is the same as that in Table 1.

Volatile Category	Compound Code	Average Relative Content (%) of Each Volatile Compounds Identified at Different Temperatures (°C)															
		JQ				YL				YH				YD			
		12	15	19	32	12	15	19	32	12	15	19	32	12	15	19	32
Alcohols	A1	1.14	1.09	0.42	0.70	4.21	4.13	2.23	1.39	0.9	2.61	2.14	-	4.91	4.31	0.66	0.64
	A2	2.03	1.59	0.76	2.58	5.42	5.38	3.17	4.75	1.01	4.4	2.63	-	11.84	9.62	1.37	2.22
	A3	26.38	24.39	12.21	10.33	28.69	28.88	31.23	8.33	62.55	56.25	57.63	14.04	15.95	16.85	15.61	28.15
	A4	-	-	-	-	-	-	-	-	-	-	-	0.38	-	-	-	-
	A5	0.48	0.36	0.25	0.56	1.28	1.3	0.79	5.7	0.2	1.06	0.62	0.49	3.3	1.92	0.52	0.54
	A6	0.29	0.26	0.82	1.59	0.17	0.11	0.12	0.72	-	2.7	-	1.41	0.46	1.67	0.52	0.17
	A7	0.14	-	-	-	0.42	-	-	-	-	-	-	-	-	-	-	-
	A8	-	-	-	-	0.07	-	0.05	-	-	-	-	-	-	0.7	-	-
	A9	1.68	0.29	2.33	-	-	-	-	-	-	-	-	-	-	-	-	-
Ketones	B1	-	-	-	-	-	-	-	-	-	-	-	-	-	-	-	-
	B2	-	0.07	-	-	-	-	-	-	-	-	-	-	0.47	-	-	-
	B3	-	-	-	-	0.05	-	0.07	-	-	-	-	-	-	-	-	-
	B4	0.14	0.2	0.27	-	-	-	0.23	0.02	-	-	-	-	-	-	-	-
	B5	3.55	1.4	2.02	2.70	4.86	3.62	4.81	36.43	0.22	2.87	1.13	15.24	1.16	2.78	2.46	3.39
	B6	7.29	3.25	11.6	5.70	7.47	8.21	12.09	10.96	1.14	2.79	2.86	4.78	4.93	6.6	8.2	5.13
	B7	0.08	0.04	0.07	0.91	-	-	0.06	0.16	-	-	0.15	0.41	-	0.19	0.31	
	B8	22.37	25.8	39.28	10.42	17.59	22.77	25.59	10.49	2.45	7.16	9.68	4.26	32.11	30.31	36.27	32.55
	B9	-	-	0.05	-	-	-	-	-	-	-	-	-	-	-	-	-
	B10	-	0.03	0.11	-	-	-	0.08	-	-	-	-	-	-	-	-	-
	B11	-	0.17	-	-	-	-	-	-	-	1.16	0.32	-	-	-	-	-
Alkenes	C1	-	0.57	-	-	-	-	-	-	-	-	-	-	-	-	-	-
	C2	19.5	31.14	4.28	2.40	10.71	13.77	9.23	1.32	-	-	1.86	-	7.94	9.4	3.72	2.07
	C3	-	-	-	-	-	-	-	-	-	-	-	0.29	-	-	-	-
	C4	-	-	-	-	-	-	-	-	0.22	1.18	-	0.21	-	-	-	-
	C5	-	-	-	-	-	-	-	-	-	-	-	-	-	-	-	-
	C6	-	0.03		0.34	2.14	0.14	-	-	-	-	-	-	-	0.17	-	-
Esters	D1	-	-	-	-		0.09	0.45	0.14	0.32	0.09	0.39	-	0.46	-	-	0.29
	D2	0.45	0.3	0.68	0.22	-	-	-	1.35	0.81	0.24	0.53	0.63	-	-	-	-
	D3	-	-	-	-	-	-	0.13	-	-	-	-	-	-	-	-	-
	D4	-	-	-	-	-	-	-	-	-	-	0.45	-	-	-	-	-
	D5	6.74	4.24	13.63	3.78	1.67	1.35	3.67	2.11	12.69	6.01	-	1.02	3.93	3.58	12.36	5.6
Alkanes	E1	-	-	-	1.89	-	-	-	-	-	-	-	-	-	-	-	-
	E2	-	-	-	-	-	-	-	-	-	-	-	-	-	-	-	-
	E3	0.12	0.14	0.37	-	0.2	0.34	0.17	0.7	0.12	0.54	0.51	1.79	1.62	0.42	0.33	0.5
	E4	-	-	-	-	0.15	0.21	0.11	0.35	-	-	-	0.51	-	-	0.26	-
	E5	-	-	-	-	-	-	-	-	-	-	-	0.32	-	-	0.07	-
	E6	0.34	-	0.66	5.52	-	-	0.27	0.52	-	-	-	4.53	-	0.24	0.77	-
	E7	0.26	-	0.28	-	0.06	0.07	0.03	0.11	0.09	0.13	-	0.3	-	-	-	-
	E8	-	-	0.08	-	-	-	-	-	-	-	-	-	-	-	0.37	-
	E9	-	-	-	-	-	-	-	-	-	-	-	-	1.2	0.23	1.23	0.35
	E10	0.62	0.2	0.66	-	0.37	0.35	0.38	0.83	0.41	0.68	0.41	1.29	0.94	0.37	0.96	1.46
	E11	0.34	0.14	0.41	1.75	-	-	0.36	1.02	0.17	0.59	0.43	1.81	0.95	0.34	0.68	0.59
	E12	-	-	-	-	-	-	0.05	0.17	0.16	0.09	-	-	-	-	-	-
	E13	0.27	0.06	0.21	1.75	0.13	0.2	0.18	0.38	0.23	0.45	0.35	0.55	1.37	0.45	0.08	0.14
	E14	0.05	0.18	-	2.76	-	-	0.18	0.93	0.14	0.11	0.76	1.19	0.97	0.14	0.59	-
	E15	0.08	0.13	0.14	1.36	-	-	-	-	0.18	0.45	0.2	1.52	-	-	0.03	-
	E16	0.25	0.19		-	-	-	-	-	-	-	-	-	-	-	0.25	-
	E17	0.36	0.11	0.31	2.56	1.91	0.49	0.29	0.36	0.46	0.85	0.76	4.14	0.59	0.24	0.48	0.49
	E18	-	-	-	-	-	-	-	-	-	-	-	-	-	0.16	0.08	-
	E19	-	-	1.29	-	-	1.21	0.36	-	-	-	-	-	-	0.33	1.34	0.7
	E20	-	-	-	6.06	1.58	0.18	0.37	-	-	-	0.97	9.84	-	-	-	-
	E21	-	-	-	-	-	-	-	-	-	0.08	0.14	0.32	-	-	-	-
	E22	0.72	0.52	1.18	6.64	-	-	-	-	3.67	1.14	0.18	8.99	-	0.36	1.63	-

**Table 3:** Numbers of chemical compounds identified in floral scent emitted from four different cultivars of *Osmanthus fragrans* at different levels of temperature.

Volatile Category	Number of Chemical Compounds Identified at Different Temperatures (°C)															
	JQ				YL				YH				YD			
	12	15	19	32	12	15	19	32	12	15	19	32	12	15	19	32
Alcohols	7	6	6	5	7	5	6	5	4	5	4	4	5	6	5	5
Ketones	5	8	7	4	4	3	7	5	3	4	5	4	4	4	4	3
Alkenes	1	3	1	2	2	2	1	1	1	1	1	2	1	2	1	1
Esters	2	2	2	2	1	2	3	3	3	3	3	2	2	1	1	2
Alkanes	11	9	11	9	7	8	12	10	10	11	10	14	7	11	16	7
Total	26	28	27	22	21	20	29	24	21	24	23	26	19	24	27	18
